# Relationship between Corneal Temperature and Intraocular Pressure in Healthy Individuals: A Clinical Thermographic Analysis

**DOI:** 10.1155/2016/3076031

**Published:** 2016-01-20

**Authors:** Claudia Fabiani, Roberto Li Voti, Dario Rusciano, Maria Giulia Mutolo, Nicola Pescosolido

**Affiliations:** ^1^Department of Ophthalmology, Humanitas Research Hospital, Via Alessandro Manzoni 56, Rozzano, 20089 Milan, Italy; ^2^SBAI Department, Sapienza University of Rome, Via A. Scarpa 16, 00161 Rome, Italy; ^3^Sooft Italia, Via Salvatore Quasimodo 136, 00144 Rome, Italy; ^4^Faculty of Medicine and Dentistry, Sant'Andrea Hospital, Sapienza University of Rome, Via di Grottarossa 1037, 00189 Rome, Italy; ^5^Faculty of Medicine and Dentistry, Sapienza University of Rome, Policlinic Umberto I, Viale Regina Elena 287/A, 00161 Rome, Italy

## Abstract

*Purpose*. To study the geographical distribution of corneal temperature (CT) and its influence on the intraocular pressure (IOP) of healthy human volunteers.* Materials and Methods*. Fifteen subjects (7 M, 8 F), 33.8 ± 17.4 years old, were enrolled in this pilot, cross-sectional study. Measurements of CT were taken after one hour with closed eyelids (CET) or closed eyelids with a cooling mask (cm-CET) and compared to baseline.* Results*. If compared to baseline, after CET, average CT significantly increased by 0.56°C in the RE and by 0.48°C in the LE (*p* < 0.001) and IOP concomitantly significantly increased by 1.13 mmHg and 1.46 mmHg, respectively, in each eye (*p* < 0.001). After cm-CET, average CT significantly decreased by 0.11°C and 0.20°C, respectively, in the RE and LE (RE *p* = 0.04; LE *p* = 0.024), followed by a significant IOP decrease of 2.19 mmHg and 1.54 mmHg, respectively, in each eye (RE *p* < 0.001; LE *p* = 0.0019).* Conclusion*. Significant variations of CT occurred after CET and cm-CET and were directly correlated with significant differences of IOP. It can be speculated that both oxidative stress and sympathetic nerve fiber stimulation by temperature oscillations may affect the regulation of AH vortex flow and turnover, thus influencing IOP values.

## 1. Introduction

Temperature is one of the fundamental regulators of tissue metabolism [1, 2]. Interest in the temperature of the eye spans almost 130 years and the ability to measure the temperature of the eye, driven by prevailing technologies, has potential importance in both research and clinical situations, including the study of ocular physiology and pathology [3–9].

New infrared ocular thermographs allow a noncontact and nonintrusive characterization of the thermal profile across the ocular surface [10–17]. Applications have included dry eye, wearing contact lens, corneal sensitivity, and ophthalmic surgery [18–24]. In addition, some studies showed a correlation between ocular surface temperature and ocular blood flow. An increase of intraocular pressure (IOP) was found to be related to a contemporary decrease of ocular perfusion pressure and ocular temperature in monkeys [25]. A recent study on humans has also reported that eyes with ischemic central venous retinal occlusion (CRVO) have lower ocular surface temperatures than nonischemic ones [26]. Moreover, in carotid artery stenosis, the eye on the affected side has been found to have an impairment in retrobulbar hemodynamics along with a reduction in corneal temperature (CT) [26]. Thermography has also been applied to explore the role of vascular factors in the physiopathology of glaucoma and Galassi et al. have recently defined ocular surface temperature as a marker of impaired retrobulbar hemodynamics in patients with glaucoma [27].

However, to date, little work has been undertaken to determine the relationship between IOP and CT [3, 22, 28]. The variations in IOP following closed eyelid test (CET) both under normal conditions and after the administration of antioxidants have recently been investigated, leading to the conclusion that CET-induced ocular hypertension could be a response to mixed stress—oxidative and thermic—with degenerative effects on the trabecular meshwork (TM) [22, 29]. Moreover, given the known influence of ambient pressure and temperature on IOP, an underwater mask has been proposed as a provocative test in the diagnosis of primary open angle glaucoma (POAG) [30].

POAG is a multifactorial and not yet well-understood pathology [31, 32]. Ocular hypertension is one known critical risk factor for glaucomatous optic neuropathy. IOP is generated and maintained via the aqueous humor circulation system in the anterior chamber (AC) of the eye. The major factor controlling IOP is the dynamic balance between aqueous humor production in the ciliary body and its draining through so-called conventional—TM and Schlemm's canal (SC)—and uveoscleral outflow pathways [33–36].

The goal of the study here reported was to map the CT in different areas of the cornea of healthy human volunteers and follow its variations after CET at room temperature or with a cooling mask (cm-CET) and correlate such variations to IOP values. The application of a physical model was then used to validate the experimental results.

## 2. Patients and Methods

This pilot, prospective, cross-sectional study was conducted following the tenets of the Declaration of Helsinki. Fifteen healthy Caucasian human volunteers (8 females and 7 males) were enrolled. The mean age was 33.8 ± 17.4 years (range 18–72). After signing an informed consent, each study participant underwent a complete ophthalmological examination, including a medical history review, best-corrected visual acuity measurement (BCVA), slit-lamp biomicroscopy, dry eye tests, and dilated fundus examination. All subjects were free from ocular and systemic diseases with no history of previous ocular surgery. Patients enrolled in the study were accepted if they had no signs or symptoms of ocular dryness: Ocular Protection Index (OPI: calculated as the ratio between BUT and the blinking frequency per minute) score ≥ 1 and OSDI score ≤ 12. In addition, since corneal pachymetry may influence temperature fluctuations and their determination, patients' central corneal thickness measured by ultrasonic pachymetry (Pacline compact multifeature pachymeter, Optikon 2000, Rome, Italy) had to be within normality limits, that is, 550 ± 20 microns.

Further inclusion criteria were as follows:

- IOP ≤ 21 mmHg, without any treatment.
- Refraction values between −4 and +4 spheric diopters.
- BCVA for far distance equal to 10/10.
- Normal angle structure at gonioscopy.
- Normal C/D ratio at slit-lamp examination.
- Normal SAP (30-2 SITA standard program).

### 2.1. Corneal Temperature Measurement

Precise spatiotemporal measurements of CT were captured through the latest generation infrared thermometer (Sola Electro-Optics, Shanghai, China). Since it is known that there is an uneven distribution of temperature in the cornea, the average CT was calculated based on the recordings of CTs in five different areas of the cornea (nasal, temporal, superior, inferior, and central). All patients were acclimatized to the clinical environment for at least 15 min. The room temperature was specifically set and controlled at all times at 25°C (≈77°F), humidity was maintained at 42.0%, and the average indoor illumination was maintained at 300 lux, considered a standard indoor level of illumination. Any air drafts were avoided. The measurements were performed between 9:00 and 11:00 a.m. in a seated position and under the conditions described by Mori et al. [18]: the subject blinked normally, then closed both eyes for 5 s, and then kept the eyes open for more than 10 s. The thermography device was set up 20 cm in front of the eye and the head was held steady with a frame. During that time, the subject was asked not to blink. If the subject blinked, a new measurement was performed.

Three measurements were taken consecutively during a single session for each eye and the average value was recorded. The measurements were repeated after one hour of CET and after the same test following the application of a cooling mask on the volunteers' eyelids. To avoid any operator related bias, all thermographic measurements were made by one single examiner.

### 2.2. IOP Measurements

IOP is reported as the mean value of three consecutive readings of each eye by Goldmann applanation tonometer (AT-900, Haag Streit Diagnostics, Switzerland) registered between 9:00 and 11:00 a.m. to minimize the effect of daily variations. To avoid interexaminer and intertonometer variances, all IOP measurements were taken by the same trained resident.

### 2.3. Physical Model

To simulate the temperature distribution of the human eye, we adopted the physical model developed by Karampatzakis and Samaras [37] that solves the Pennes bioheat transfer equation [38] coupled with the incompressible Navier-Stokes equation of fluid dynamics.

According to such a physical model, the eye can be modeled by seven regions with different thermal properties—the cornea, the anterior chamber, the trabecular meshwork, the iris, the lens, the vitreous humor, and the sclera. The basal metabolic heat generation, as well as the blood perfusion, mainly occurs in the iris and in the sclera. This model also includes secretory inflow, drainage, and circulation of the AH.

### 2.4. Statistical Analysis

The results obtained were statistically analyzed by Student's *t*-test for paired samples. *p* values below 0.05 were considered significant and denoted by one (^*∗*^ *p* < 0.05) or two (^*∗∗*^ *p* < 0.01) asterisks (SPSS V.19, IBM SPSS Statistics, USA).

## 3. Results

**Figure 1:**
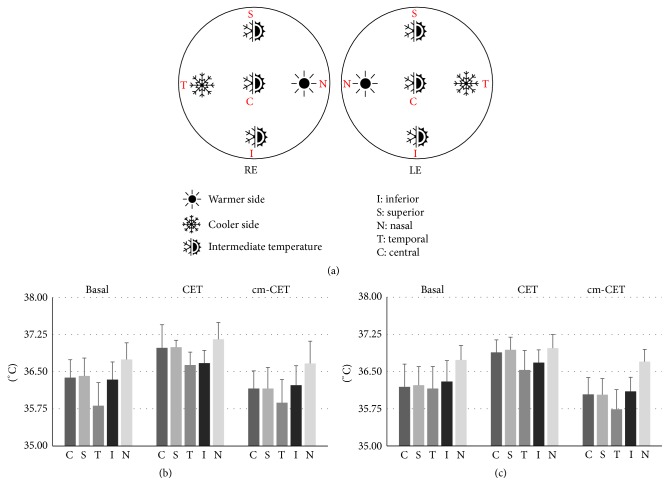
(a) A graphic illustration of the distribution of temperature on the corneal surface. (b) and (c) Values of the temperature measured in the different areas of the cornea for the right (b) and left (c) eye with open eyelids (basal); after one hour with closed eyelids (CET) and after one hour with closed eyelids wearing a cooling mask (cm-CET).

We analyzed 30 eyes of 15 healthy volunteers.  Figure 1 shows the distribution of basal values across the cornea. In each eye, the lowest temperature was observed at the temporal side, the highest temperature was observed at the nasal side, and intermediate values were observed along the corneal longitudinal axis (superior, central, and inferior). The same distribution was also maintained after CET and cm-CET.

**Figure 2:**
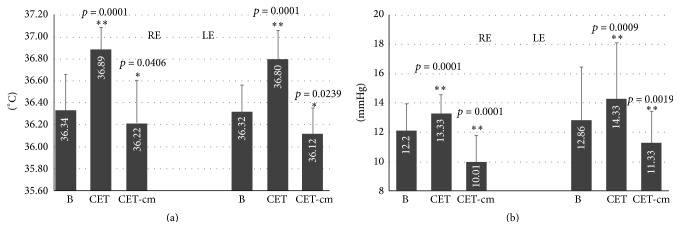
Variation of the average corneal temperature (a) and the IOP (b) in the right (RE) and left (LE) eye with open eyelids (b); after one hour with closed eyelids (CET) and after one hour with closed eyelids wearing a cooling mask (cm-CET). ^*∗*^ *p* < 0.05; ^*∗∗*^ *p* < 0.01.

CT mean basal values were 36.33 ± 0.33°C in the right eye (RE) and 36.32 ± 0.24°C in the left eye (LE). After CET, all the temperatures tended to increase with respect to the basal values (mean values: 36.89 ± 0.20°C in the RE and 36.80 ± 0.26°C in the LE) whereas after cm-CET all the temperatures showed a tendency to decrease (mean values: 36.22 ± 0.39°C in the RE and 36.12 ± 0.23°C in the LE). On average, after CET a highly significant increase of the corneal temperature of 0.56°C for the RE and 0.48°C for the LE (*p* < 0.001 for both eyes) was observed and after cm-CET there was a significant decrease of 0.11°C for the RE and 0.20°C in the LE (RE *p* = 0.04 and LE *p* = 0.024 for both eyes) (Figure 2(a)).

Correspondingly, we observed significant variations in IOP, which increased after CET by 1.13 mmHg in the RE and 1.46 mmHg in the LE (basal mean values: 12.20 ± 1.72 mmHg in the RE and 12.87 ± 3.6 mmHg in the LE; after CET mean values: 13.33 ± 1.2 mmHg in the RE and 14.33 ± 3.8 mmHg in the LE; *p* < 0.001 for both eyes), whereas it significantly decreased after cm-CET by 2.19 mmHg in the RE and by 1.54 mmHg in the LE (basal mean values: 12.20 ± 1.72 mmHg in the RE and 12.87 ± 3.6 mmHg in the LE; after cm-CET mean values: 10.01 ± 1.78 mmHg in the RE and 11.33 ± 2.1 mmHg in the LE; RE *p* < 0.001 and LE *p* = 0.0019) (Figure 2(b)).

**Figure 3:**
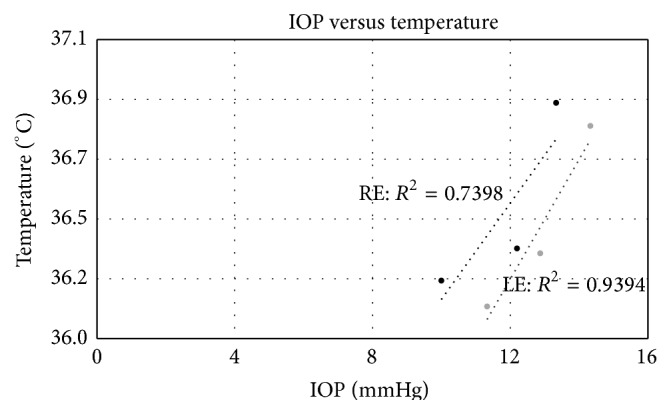
Graph of the relationship between average corneal temperature (*y*-axis) and IOP (*x*-axis) for the right eye (RE: closed, black circles) and left eye (LE: closed, grey circles). The correlation coefficient for a linear correlation is reported.

Figure 3 shows that there is indeed a direct correlation between average CT and IOP, with a correlation coefficient scoring 0.74 in the RE and 0.94 in the LE.

**Figure 4:**
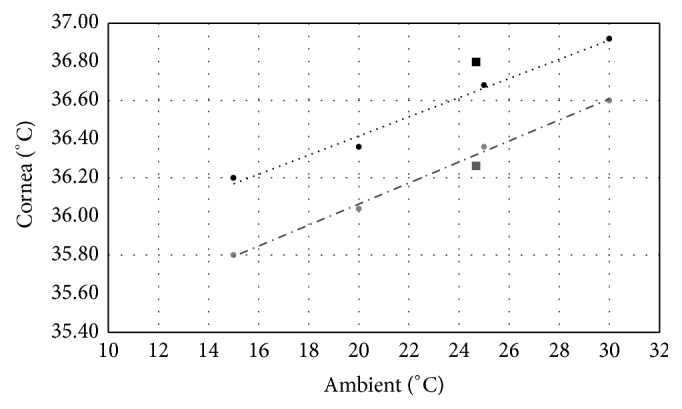
Theoretical temperature values extrapolated from Karampatzakis's physical model, relating the ambient temperature (*x*-axis) to the average corneal surface temperature (*y*-axis) with open eyelids (closed, grey circles, and dotted line), or after one hour of CET (closed, black circles, and continuous line). The closed squares represent the actual measured values at an ambient temperature of 25°C of averaged corneal temperatures for the right and left eyes with open eyelids (lower grey square), or after one hour of CET (upper black square).

Finally, the application of the physical simulation model by Karampatzakis and Samaras [37] and Pennes bioheat transfer equation [38] (Figure 4) shows the following:

- The mean temperature of the eye is influenced by the ambient temperature: it increases by about 1.0°C when the ambient temperature rises from 15°C to 30°C. The data that we obtained at an environmental temperature of 25°C were 36.3°C with open eyelids and 36.8°C after CET, in agreement with the theoretical prediction.
- The evaporation rate of the thin tear film on the cornea decreases its temperature, similarly to sweat evaporation on the skin; therefore, blocking the evaporation increases the overall surface temperature, as less heat is being exchanged between the cornea and the environment. Mathematical simulations estimate such a temperature rise at about 0.4°C, in agreement with the experimental data after CET, when the evaporation was stopped for some time, and consequently the CT increased by 0.56°C (RE) and 0.48°C (LE).

## 4. Discussion

This study shows that, in normal eyes, following CET and cm-CET, IOP significantly changes in response to variations of CT. In agreement with previous studies [34–37], we also observed an overall increase in CT after CET. In addition, our study is the first to report an overall decrease of CT after application of a cooling mask on the lid surface (cm-CET). Moreover, a direct correlation was found between CT and IOP, with IOP values following the increase or the decrease of average surface CT values. To date, the physiological link between CT and IOP still remains somewhat inconclusive. Previous investigations demonstrated that eyelid closure, or the use of an underwater mask with open eyelids, can raise IOP because of an increase of local temperature [30]. Further studies confirmed the relationship between local temperature, variation in IOP, and anterior chamber oxidative stress following CET [29, 39–41].

As previously reported, our data confirm that CT varies in response to tear film evaporation rate, which in turn is influenced by environmental temperature and blinking rate [19, 42–44]. However, our results also show a peculiar pattern of CT distribution across the corneal surface, not in line with previous findings [12, 14, 15, 45]. In fact, in all experimental settings (basal, after CET, and cm-CET), we detected the coolest area at the corneal temporal region while the warmest area was at the nasal side. Along the corneal vertical axis, the temperatures showed intermediate values. To explain and validate our data, the physical model by Karampatzakis and Samaras [37] and Pennes bioheat transfer equation [38] was applied and allowed us to conclude that AH flow depends on CT distribution. Numerical simulations show that the circulation of AH follows the temperature difference between the temporal and nasal side of the cornea and that an increase of AH flow from stagnation to fast vortices correlates with the increasing asymmetry between temporal and nasal temperatures, thus influencing the balance between production and outflow, and finally IOP values. Accordingly, CT differences between nasal and temporal sides were higher after cm-CET (0.80 and 0.96 for the RE and LE, resp.) than after CET (0.52 and 0.44 for the RE and LE, resp.). This may indeed suggest a faster flow with lower temperatures, favoring a higher discharge of AH, and therefore a lower IOP.

IOP depends on the dynamics of AH turnover, which in turn is a balance between secretion and excretion. Three mechanisms are involved in AH formation by the ciliary body: diffusion, ultrafiltration, and active secretion, the last being the major contributor to its formation. AH can be considered analogous to a blood surrogate as it provides nutrition, removes excretory products from metabolism, transports neurotransmitters, stabilizes the ocular structure, and contributes to the regulation of the homeostasis of the surrounding ocular tissues [33–36]. AH, as part of a vascular circulatory loop, returns to the blood flow by passing through the TM, a uniquely modified vessel wall interposed between the anterior chamber and SC or through the uveoscleral pathway [34, 36].

Our results suggest that temperature may affect AH balance through the regulation of its secretion, excretion, and flow dynamics. Secretion is enhanced by increasing temperatures, because of increased blood flow in the anterior segment of the eye due to vasodilation and upregulation of metabolic processes in the ciliary body. The opposite effect is therefore expected after decreasing the temperature, since it is known that low temperatures stimulate the sympathetic system inducing vasoconstriction. Excretion is also expected to be regulated by temperature. At high temperatures, there is an increase of oxidative stress and inflammatory processes due to increased metabolic activities. These events may negatively affect the draining through the TM, since it has been shown that high temperature increases the cellularity of the anterior chamber angle structures [33, 34]. The opposite is expected with low temperatures.

The application of the physical model by Karampatzakis and Samaras [37] confirmed the direct correlation between IOP and CT and the influence of tear film evaporation and environmental temperature on CT. Moreover, and more interestingly, such numerical simulation suggested that AH responds to increasing surface temperature generating vortices that might contribute to the variations in IOP observed in our experiments.

Finally, the presence of several transient receptor potential (TRP) channel isotypes that are responsive to temperature variations in the three corneal layers and in corneal nerve fibers [46] may suggest an involvement of this receptor type also in controlling the aqueous humor dynamics, although no reports exist yet indicating such kind of interaction.

IOP is the only treatable risk factor in glaucoma, one of the world's leading causes of blindness. The existing correlation between CT and IOP suggests that CT may affect short-term IOP control and its fluctuations. Such IOP fluctuations are related to faster glaucoma progression. Therefore, controlling IOP and its fluctuations is a main therapeutic goal in glaucoma treatment. Temperature and oxidative stress remain additional targets in the control of glaucoma progression [29, 31, 34, 36, 40]. It will be interesting to extend these findings studying the influence of temperature on IOP not only in the seated position but also in the supine position and on a larger population. Moreover, it could be interesting to see whether the same correlation persists in the presence of ocular hypertension. In such case, the use of a cooling mask during the night, when peaks of IOP are mostly expected with negative effects on POAG progression, might be advisable.
